# Intestinal microbiota composition of children with glycogen storage Type I patients

**DOI:** 10.1038/s41430-024-01412-0

**Published:** 2024-02-24

**Authors:** Sabire Gokalp, Ener Cagri Dinleyici, Cansu Muluk, Asli Inci, Emine Aktas, Ilyas Okur, Fatih Ezgu, Leyla Tumer

**Affiliations:** 1 Gazi University Faculty of Medicine, Department of Pediatric Nutrition and Metabolism, Ankara, Turkey; 2 Eskisehir Osmangazi University Faculty of Medicine, Department of Pediatrics, Eskisehir, Turkey; 3 Gazi University Faculty of Medicine, Department of Pediatrics, Ankara, Turkey

**Keywords:** Metabolic disorders, Bacteria

## Abstract

**Aim:**

Dietary therapy of glycogen storage disease I (GSD I) is based on frequent feeding, with a high intake of complex carbohydrates (supplied by uncooked cornstarch), restriction of sugars, and a lower amount of lipids. There is limited information about the dietary regimen in patients with GSD, which might affect the intestinal luminal pH and microbiota composition. The aim of this study to investigate the intestinal microbiota composition in patients with GSD receiving diet treatment.

**Method:**

Twelve patients who were followed up with GSD I after the diagnosis receiving diet therapy and 11 healthy children have been enrolled. Intestinal microbiota composition was evaluated by 16 s rRNA gene sequencing.

**Results:**

A significant difference was found for beta-diversity between the GSD group and controls. A significantly lower abundance of Firmicutes and higher abundance of Actinobacteria was found in GSD group compared to the controls. *Akkermansia, Pseudoalteromonas, Uruburella, and Castellaniella* were dominant in the GSD patients at the genus level, while *Faecalibacterium, Bacterioides, Gemmiger, Parabacteroides* in the control group. At species level, *Faecalibacterium prausnitzii* decreased, and *Akkermansia muciniphila* were dominant in children with GSD.

**Discussion:**

There is a substantial change in the composition of the gut microbiota, reduction of *F. prausnitzii* and an increase of *A. muciniphila* in children with GSD receiving consumption of uncooked cornstarch. Alterations of the intestinal microbiota might be related with the disease itself or dietary restrictions in patients with GSD, however, in certain condition, dysbiosis can negatively affect the course and make it difficult to control the disease.

## Introduction

Glycogen storage disease Type I (GSD I, von Gierke’s disease, OMIM 232200) is an autosomal recessive disorder. There are two types of GSD I, which are resulting from deficiencies of glucose-6-phosphatase (GSD Ia, about 80% of patients) and glucose-6-phosphate transporter (GSD Ib). Hypoglycemia symptoms, hepatomegaly, poor growth, short stature and distended abdomen may be occur. Glucose-6-phosphate can not be converted to glucose and hypoglycemia occurs. Fasting hypoglycemia is the main metabolic abnormality. Other metabolic abnormalities are lactic acidosis, hyperuricemia, hypertriglyceridemia and hypercholesterolemia. Many patients with GSD I will develop different complications such as liver adenomas, chronic kidney disease, urolithiasis, osteoporosis, and anemia. Poor metabolic control causes these complications. Neutropenia and inflammatory bowel disease (IBD) are characteristic features or complications of GSD Ib [1–3]. Dietary treatment is the main part of GSDI therapy. A regular carbohydrate intake is necessary to prevent hypoglycemia and to provide good metabolic control. Main approaches are ingestion of slowly digested carbohydrates, usually uncooked cornstarch (UCCS) and feeding at nighttime oral or continuous tube (especially in children) [4–6]. Modified form of corn starch with a different content of amylopectin and resistant starch can provide normoglycemia during nighttime in some patients [7, 8]. Glucose requirements generally decrease with age, and adults typically have a longer fasting tolerance compared to children, which facilitates overnight glucose control [9].

Few studies available in the current literature report the composition of gut microbiota of GSD I in relation to diet composition [10, 11]. Colonetti et al. [11] examined the gut microbiota of GSD patients while also taking diet into careful consideration as a crucial determinant that determines the microbiota composition. Dietary regimens in GSD might alter the pH of the luminal fluid and produce structural changes to the microbiota of the intestinal tract. Because of the lower pH, both the production of bacteria that ferment fiber and the amount of SCFAs in the gut are decreased. The decreased level of SCFAs may affect inflammatory status of gut in GSD. There was a general trend toward increased abundance of *Actinobacteria, Proteobacteria, and Escherchia/Shigella* in patients with GSDs, while there was a general trend toward decreased abundance of *Euryarchaeota, Coprococcus, Blautia, Anaerostipes, Odoribacter, and Faecalibacterium*. Taxonomic shifts, which were associated with dysbiosis and occurred in individuals with GSD and reduced microbial diversity, were seen in these patients [10, 11]. The dietary alteration that was utilized to treat the condition was mostly responsible for the dysbiosis that was observed [11]. The purpose of this study was to investigate the microbiota composition in patients with GSD and determine how it is related to diet.

## Patients and Method

This study was a cross-sectional, observational study among patients with GSD Type Ia and Ib which were followed-up from the outpatient clinic of the Gazi University Faculty of Medicine, Department of Pediatric Nutrition and Metabolism. All patients were between 3–18 years old. Exclusion criterias were the presence of obesity, diabetes mellitus, autism, asthma, autoimmune diseases, receiving proton pump inhibitors, probiotic or antibiotic therapy in last three months, presence of gastrointestinal surgical history. Age and sex matched healthy children served as control group. The study was carried out with the approval number 253 of Gazi University Medical Faculty Ethics Committee dated 04 March 2021.

### Nutritional assessment

Dietary intakes were recorded by asking the parents. A three-day food consumption record was taken, with one day of the recording coinciding with the weekend. The results were analyzed using the Computer Assisted Nutrition Program developed for Turkey, the Nutrition Computer Package Program (BEBIS) [12]. Regular treatment was given to patients and their parents was questioned for dietary inconsistency. Three days of dietary consumption and nutrients were analyzed. The nutrient composition of the diet was 60–70% calories from carbohydrates, 10–15% calories from protein, and from fat ( < 30% for children older than 2 years). Sucrose (fructose and glucose) and lactose (galactose and glucose) were avoided from the diet To prevent hypoglycemia, patients with GSD I were given UCCS 8–12 times a day, on average. Dosing of UCCS include 1.6 g of UCCS per kilogram and 1.7–2.5 UCCS per kilogram for older children, adolescents [2]. Nutritional assessments did not include multivitamin intake or other dietary drugs, which may have influenced the composition of the gut microbiota. Records of medications use were kept.

### Sample collection, fecal DNA extraction, sequencing, and bioinformatics analysis

Fresh stool samples were collected from patient and control group at any time of day and stored upright in a 15 ml Falcon tube at −80 °C until DNA extraction. QuickGene (Kurabo, Japan) was used to extract DNA from the stool samples. First, 25 mg of each stool sample was transferred to a homogenization tube with 250 µl of tissue lysis (MDT) solution. To homogenize the solution, 15 mg of 0.1 mmø glass beads or 10 1.0 mmø zirconia beads were added to the tube and then homogenized for 2 × 120 s at 5000 rpm. After the sample was homogenized, 25 µl of Proteinase K (EDT) solution was added and incubated at 56 °C for 60 min. The tube was then centrifuged at 15,000 g for 10 min at room temperature. After centrifugation, 200 µl of supernatant was transferred to a 1.5 mL microtube. Then 180 µl of cell lysis (LDT) solution was added and vortexed for 15 s. The microtube was left to incubate at 70 °C for 10 min. In the next step, 240 µl of 99% cold ethanol was added and vortexed for 15 s. The entire contents of the microtube were transferred to a QuickGene (Kurabo, Japan) filtered cassette, where washes and elutions were performed following the instrument protocol. Three washes were performed using 750 μl of wash buffer (WDT) solution. Based on the results of the extraction process, bacterial 16 S ribosomal RNA (rRNA) gene target sequencing was performed from the materials obtained in the study. The resulting genomic DNA was amplified using 16 S V3-V4 314F-860R primer sets, and library preparation was performed using a Nextera XT DNA library preparation kit and indices (Illumina, CA, USA). The amplicon library was cleaned by selecting large fragments (AMPure XP, Beckman Coulter). It was then normalized and aggregated. After the library was prepared, the NovoSeq 6000 (Illumina, CA, USA) instrument was used to run the sequencing.

Pair-ended Illumina reads (2 × 250) were transferred to the QIIME2 environment [13]. All samples had a sequence depth greater than 100X, and no samples were omitted from the run. Quality clipping, chimera detection, and read cleaning were implemented using the QIIME2 Dada2 pipeline (via q2‐dada2) [14]. Amplicon sequence variants (ASV) generated by Dada2 were mapped to the GreenGenes (http://greengenes.lbl.gov) database [15, 16]. The phyloseq object was created from qiime2 artifact files in the R 4.1 environment [17, 18]. Alpha diversity metrics were calculated from the phyloseq object using the microbiome R package. Significant differences between groups were calculated using the Kruskal–Wallis rank sum test. Beta diversity was computed by phyloseq, including the Bray-Curtis, Jaccard, Unweighted UniFrac, and weighted UniFrac distance metrics. Beta diversity statistical significance between groups was calculated using a PERMANOVA test via the Adonis function in the vegan R package. Intergroup *p* values were calculated using the Kruskal–Wallis test. Specific differences between groups were determined by differential abundance analysis using the Deseq2 R package [19]. Linear discriminant analysis effect size (LEFSe) analyses were performed between groups to determine statistically significant taxonomies [20].

## Results

The study involved 11 GSD Ia patients (91.6%) and one GSD Ib patient (8.5%) (four boys and eight girls). The mean age of these patients was 7.3-month-old (min:1 month-max:36 months) when they were diagnosed. Patients were treated with special diet since first diagnosed. Ages ranged from eight to 16 years old. A total of nine patients received allopurinol, one received filgrastim, and 12 received triglyceride-lowering medications. GSD 1b patient had neither neutropenia nor IBD in this study. Anthropometric measurement of patients was shown in Table 1. Three patients had short stature and two patients were underweight. The dietary intakes and macronutrient compositions of patients are reported in Table 1. They have higher carbohydrate intakes than healthy age matched population. Protein intakes are similar with healthy age matched population. Uncooked corn starch intakes are higher also.

**Table 1:** Anthropometric measurements and nutritional intakes of 12 children with GSD.

Patient	Age (year)	Gender (B/G)	Height (cm)	Height percentile	Weight (kg)	Weight percentile	Energy(kcal)	Protein (g) (energy %)	Fat (g) (energy %)	Carbohyrate (g) (energy %)	Uncooked cornstarch (g/day)
1	16	B	150	3	48	3	2026,9	47,8 g (10%)	40,6 g (18%)	360,8 g (73%)	150 g
2	8	G	134	2	23	16	2541,6	58,1 g (9%)	57,4 g (20%)	440,5 g (71%)	159 g
3	12	G	142	15	53	90	1584,3	52,6 g (14%)	57,4 g (32%)	211,8 g (54%)	135 g
4	8	G	106	5	22	10	2638,3	62,1 g (10%)	62,4 g (20%)	450,5 g (70%)	202 g
5	5	G	110	35	21	70	1344,3	25,7 g (8%)	32,8 g (22%)	232,9 g (71%)	119 g
6	6	B	111	6	21	39	2635,0	56,0 g (9%)	119,4 g (40%)	332,2 g (51%)	121 g
7	7	G	122	37	34	90	1975,2	45,5 g (9%)	58,4 g (26%)	313,4 g (64%)	104 g
8	10	G	130	4	41	77	1627,1	39,0 g (10%)	76,5 g (42%)	191,4 g (48%)	210 g
9	12	G	168	81	67	89	3215,0	81,8 g (10%)	106,8 g (29%)	475,1 g (60%)	230 g
10	13	B	134	3	38	5	1873,2	40,0 g (9%)	62,1 g (30%)	280,0 g (62%)	90 g
11	12	B	109	5	19	2	1950	45 g (11%)	63 g (31%)	275 g (58%)	88 g
12	7	G	123	45	34	90	1900	40 g (10%)	57 g (25%)	315 g (65%)	112 g

### Intestinal microbiota analysis

**Fig. 1:**
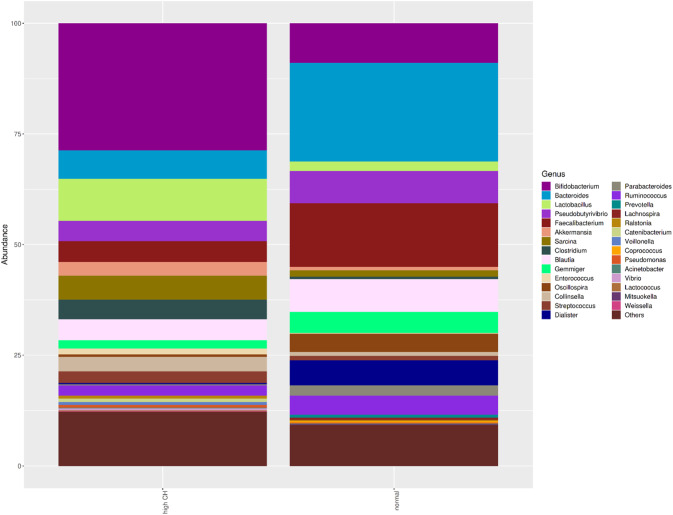
Intestinal microbiota composition of the study groups at genus levels. Bacterial community relative abundance analysis at the genus (relative abundance >1%; bacteria with relative abundances <1% were pooled in the ‘others’ category and sorted by total concentration).

Alpha diversity refers to the diversity within the sample or grouped data. In our study, regarding to alpha diversity parameters, there was no difference in observed OTU, Chao1, Shannon and Simpson indices between two groups (*p* > 0.05). A statistical difference was found between two groups in the results of Bray Curtis (A), Jaccard (B), Weighted Unifrac (C) and Unweighted Unifrac (D) baseline coordinate analysis (PCoA) in stool samples (*p* = 0.001, *p* < 0.01, *p* < 0.01, *p* < 0.05). At the phylum level of GSD patients, a change was found in the *Actinobacteria* phylum, (23.9%). In this group, *Firmicutes* (51.4%), *Bacteroidetes* (5.4%), *Proteobacteria* (16.8%) were detected. A comparison of the microbiota composition at the genus level between GSD patients and healthy controls showed in Fig. 1.

**Fig. 2:**
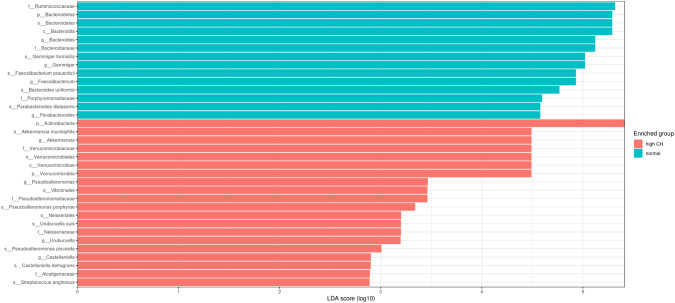
LEfSe analysis grafics showing bacterial taxa that were significantly different in abundance between GSD group and healthy controls. LefSe analysis was performed to identify differentially abundant taxa for which the LDA scores are shown. Only species and functional modules with LDA effect size >2 and FDR-corrected *p* value < 0.05 were plotted. Horizontal bars represent log.10 converted LDA score indicated by vertical dotted lines. Different colors represent different groups. p—phylum, c-class, o—order, f—family, g—genus, s—species. Red: High CH (GSD group receiving diet). Green: Normal, Healthy children.

LEfSe (linear discriminant analysis effect size) analysis (LDA threshold value > 2, *p* < 0.05) was used to determine significant bacterial compositions between two groups. The LEfSe analysis of GSD patients and healthy controls is given in Fig. 2.

*Akkermansia, Pseudoalteromonas, Uruburella, and Castellaniella* were dominant in the GSD patients at the genus level, while *Faecalibacterium, Bacterioides, Gemmiger, Parabacteroides*, which were the dominant genera, were found in the control group. The dominant species were *Faecalibacterium prausnitzii, Gemmiger formicilis, Bacteroides uniformis, Parabacteroides distasonis* in the control group. *Akkermansia muciniphila, Pseudoalteromonas porphyrae, Uruburuella suis, Pseudoalteromonas piscicida, Castellaniella defragans* and *Streptococcus anginosus* were predominant species level in GSD patients.

## Discussion

Our study investigated effect of UCCS-rich diet on gut microbiota of GSD patients. We showed changed intestinal microbiota composition, lower abundance of *Firmicutes* and higher abundance of *Actinobacteria* in GSD patients compared to control group. Firmicutes, specifically *Faecalibacterium prausnitzii* and *Clostridium leptum* of the family *Ruminococcaceae, Eubacterium rectale* and *Roseburia spp*. of the family *Lachnospiraceae*, are the primary butyrate-producing bacteria in the human gut [21, 22]. Short chain fatty acids, especially butyrate is important for human intestinal health. Luminal butyrate production can protect intestinal barrier function, immune system regulation and balance of gut microbiota. Butyrate-producing bacteria fermented undigested carbohydrates in the intestinal lumen and produce SCFAs. Acidic lumen provided by butyrate-producing bacteria balances microbiota and gut [20]. Their balance has positive effect on gut and improves digestion and immune system. If there is an imbalance, dysbiois can start [23]. Colonic epithelial cells which provide normal luminal barrier function use energy from lumen directly. More than 90% of luminal SCFAs are produced in lumen and absorbed by luminal epithelial cells. If SCFAs are decreased in lumen, colonic epithelial cells have nutritional deficiency and atrophy [21]. Abundance of butyrate-producing bacteria decrease SCFAs in the lumen and cause dysbiosis. In our study, *Firmicutes* were decreased, especially *Faecalibacterium prausnitzii*. Reduced *F. prausnitzii* has been reported in cystic fibrosis, inflammatory bowel disease, hypertension, multiple sclerosis, obesity, type 2 diabetes, rheumatoid arthritis, celiac disease, and in children with COVID-19 and MIS-C [24]. These conditions could all be considered to have gastrointestinal tract inflammation. In our research, we also discovered a decrease in F. prausnitzii in the intestinal microbiota composition of the GSD individuals, which is suggestive of gastrointestinal system involvement or dysbiosis in GSD cases. There are a few studies which were reported gut microbiota of GSD patients [10, 11]. Colonetti and coworkers [11]. analyzed gut microbiota of GSD patients with different types. They suggested that the UCCS riched diet can lower fecal pH and low fecal pH provides environmental selection factor to the bacteria in the lumen, so dysbiosis occurs. They found that the *Actinobacteria* and *Proteobacteria* phylum were increased in GSD patients while the *Euryarchaeota* was decreased. The microbiome of GSD patients showed low diversity and was dominated by *Escherichia/Shigella* [11]. They showed that the phylum *Proteobacteria* was more abundant in GSD patients than in controls. This result is as same as our study. *Proteobacteria* is a gram-negative phylum with an outer membrane composed of lipopolysaccharides, which are related to inflammation. Higher levels of *Proteobacteria* can be a strong marker of dysbiosis [25].

In our study, we showed increased abundance of *Akkermansia muciniphila*. *A. muciniphila*, an intestinal mucin-degrading anaerobe bacteria that is considered currently as a therapeutic microbe [26]. *A. muciniphila* belongs to the phylum Verrucomicrobia, is a gram-negative bacterium, and composes 1–5% of the total human microbiome [27]. *Akkermansia muciniphila* derives mucin and uses carbohydrates. Mucin is composed of fucose, galactose, N-acetylgalactosamine and N-acetylglucosamine. Mucin is an energy source for mucin-degrading bacteria such as *A. muciniphila* [28, 29]. *Akkermansia muciniphila* is also produces propionate and acetate as SCFAs. It may be suggested that high carbohydrate diet increased *A. muciniphila* and protected gut. But dysbiosis has many components and mucin is not only part of gut. There are several factors that affect the abundance of *A. muciniphila* in the gut. The dietary interventions which investigated the abundance of *A. muciniphila* were caloric restriction diet, reduced energy diet, fermentable oligo-, di-, mono-saccharides and polyols (FODMAP) low diet, supplemental fibres, sodium butyrate and inulin, pomegranate extract, kiwifruit capsules, and resveratrol [30]. *Akkermansia muciniphila* is less abundant in high-fat and poor fiber diet [31]. Dao and coworkers analyzed a caloric restriction diet compared to a weight stabilization diet between overweight and obese. In the weight stabilization diet group, the abundance of *A. muciniphila* decreased in participants with both low and high baseline levels of *A. muciniphila*. In the caloric restricted group, the abundance of *A. muciniphila* decreased in participants with a high baseline level of *A. muciniphila* and increased in participants with low *A. muciniphila* levels [32]. Another study compared placebo and reduced energy diet in patients with Type 2 diabetes. They showed that reduced-energy diet increased levels of *A. muciniphila* by 125% [33]. Our GSD I patients treated with UCCS riched diet. We know that gut and probiotic bacteria and diet ingredients such as carbohydrates could increase the abundance of *A. muciniphila* [31]. In correlation with this study, we found increased abundance of *A. muciniphila*.

Our study has some limitations. All patients involved in the study were individuals diagnosed with GSD Type 1 and were treated with dietary interventions. Alterations of the microbiota composition of these patients might be related disease itself and/or dietary intervention during the sampling period. The composition of the microbiome was not analyzed at the time of GSD diagnosis. However, all of these cases suggest an alteration of microbiota composition, as dietary intervention is obligatory for individuals with GSD. It would be also useful to evaluate and calculate the dietary content of the control group in future studies. Age range of patients with GSD was very wide and might affect the microbiota composition. There is only one patient with GSD Type 1b, and previous studies showed that these patients have a potential risk for IBD (while this case have no IBD). Further large studies including different age groups and different subtypes of GSD Type 1 would help to understand the trajectory of microbiota composition.

The investigation of gut microbiome is essential for lightening the role of microbial factors on inherited metabolic diseases. Microbiota can be affected by pH and inflammation, and the differences in these factors between GSD I and control groups may be related to UCCS riched diet or genetic. Several bacterial taxa were different in GSD patients than in controls, and those groups are consistent with the complications of GSD I. The microbiome compositions of GSD I patients may be altered by immune metabolic pathways of genetic impairment, and may be affected from individual response to treatment [11]. Further long term follow up study highlight the effect of disease or treatment modalities on gut microbiota composition in children with GSD. In some circumstances, dysbiosis can adversely affect the course of inherited metabolic illnesses and make it harder to regulate the disease. Alterations of the intestinal microbiota may be related to the disease itself or dietary limitations in patients with GSD I.

## Data Availability

Authors can share manuscript data if requested.
