# Correction: Intestinal microbiota composition of children with glycogen storage Type I patients

**DOI:** 10.1038/s41430-024-01533-6

**Published:** 2024-10-31

**Authors:** Sabire Gokalp, Ener Cagri Dinleyici, Cansu Muluk, Asli Inci, Emine Aktas, Ilyas Okur, Fatih Ezgu, Leyla Tumer

**Affiliations:** 1 Gazi University Faculty of Medicine, Department of Pediatric Nutrition and Metabolism, Ankara, Turkey; 2 Eskisehir Osmangazi University Faculty of Medicine, Department of Pediatrics, Eskisehir, Turkey; 3 Gazi University Faculty of Medicine, Department of Pediatrics, Ankara, Turkey

**Keywords:** Metabolic disorders, Bacteria

Correction to: *European Journal of Clinical Nutrition* 10.1038/s41430-024-01412-0, published online 24 February 2024

The following sentence was added to the Funding section: “The study was supported by the Gazi University Scientific Research Projects Department (Gazi University - TGA-2021-7036).” Additionally, the following Acknowledgements section was included: “The authors appreciate the Gazi University Scientific Research Projects Department for their financial support.” The original article has been corrected.

